# Kinetic Characterization of PB1-F2-Mediated Immunopathology during Highly Pathogenic Avian H5N1 Influenza Virus Infection

**DOI:** 10.1371/journal.pone.0057894

**Published:** 2013-03-01

**Authors:** Olivier Leymarie, Grégory Jouvion, Pierre-Louis Hervé, Christophe Chevalier, Valérie Lorin, Jérôme Lecardonnel, Bruno Da Costa, Bernard Delmas, Nicolas Escriou, Ronan Le Goffic

**Affiliations:** 1 Unité de Virologie et Immunologie Moléculaires, UR 892 INRA, Domaine de Vilvert, Jouy-en-Josas, France; 2 Institut Pasteur, Unité Histopathologie Humaine et Modèles Animaux, Département Infection et Epidémiologie, Paris, France; 3 Institut Pasteur, Unité de Génétique Moléculaire des Virus à ARN, Département de Virologie, Paris, France; 4 CNRS, URA30I5, Paris, France; 5 Univ. Paris Diderot, Sorbonne, Paris Cité, EA 302, Paris, France; 6 Centre de Ressources Biologiques pour la Génomique des Animaux Domestiques et d'Intérêt Economique, CRB GADIE INRA, Domaine de Vilvert, Jouy-en-Josas, France; Centre of Influenza Research, The University of Hong Kong

## Abstract

The PB1-F2 protein encoded by influenza A viruses can contribute to virulence, a feature that is dependent of its sequence polymorphism. Whereas PB1-F2 from some H1N1 viruses were shown to exacerbate the inflammatory response within the airways, the contribution of PB1-F2 to highly pathogenic avian influenza virus (HPAIV) virulence in mammals remains poorly described. Using a H5N1 HPAIV strain isolated from duck and its PB1-F2 knocked-out mutant, we characterized the dynamics of PB1-F2-associated host response in a murine model of lethal pneumonia. The mean time of death was 10 days for the two viruses, allowing us to perform global transcriptomic analyses and detailed histological investigations of the infected lungs at multiple time points. At day 2 post-infection (pi), while no histopathological lesion was observed, PB1-F2 expression resulted in a significant inhibition of cellular pathways involved in macrophage activation and in a transcriptomic signature suggesting that it promotes damage to the epithelial barrier. At day 4 pi, the gene profile associated with PB1-F2 expression revealed dysfunctions in NK cells activity. At day 8 pi, PB1-F2 expression was strongly associated with increased transcription of genes encoding chemokines and cytokines implicated in the recruitment of granulocytes, as well as expression of a number of genes encoding enzymes expressed by neutrophils. These transcriptomic data were fully supported by the histopathological analysis of the mice lungs which evidenced more severe inflammatory lesions and enhanced recruitment of neutrophils in the context of PB1-F2 expression, and thus provided a functional corroboration to the insight obtained in this work. In summary, our study shows that PB1-F2 of H5N1 HPAIV markedly influences the expression of the host transcriptome in a different way than its H1N1 counterparts: H5N1 PB1-F2 first delays the initial immune response but increases the pulmonary inflammatory response during the late stages of infection.

## Introduction

Highly pathogenic avian influenza virus (HPAIV) infections result in case-fatality rates of up to 60% in humans, making this pathology one of the most severe infectious respiratory disease [1]. Although human-to-human transmission of HPAIV is a very rare event [2], the genetic reassortment of a HPAIV with seasonal influenza A viruses (IAV) could give rise to a particularly virulent strain with pandemic capacities [3]. The emergence of such a virus is therefore a global health concern.

A good understanding of mechanisms that lead to HPAIV-induced fatal outcome could help to design new therapy against this pathogen. However the pathogenesis mediated by HPAIV is complex and several differences contrast with seasonal IAV including dissemination beyond the respiratory tract, higher viral cytolytic damages, differences in the tissue tropism and differences in the host response intensity [4].

Among the multiple parameters that are responsible for the development of a severe disease, the deregulation of the host response is suspected to be a critical element implicated in the pathogenesis. Studies on patients suffering from HPAIV infections have described an excessive immune reaction called “cytokine storm” that led to an acute respiratory distress syndrome [5]. The viral factors contributing to this hypercytokinemia are mainly NS1, NA and HA [6], [7], [8], [9], but more recently the small accessory protein PB1-F2 has also been described to contribute to the viral pathogenesis of IAV [10], [11], [12] and H5N1 HPAIV [13], [14].

PB1-F2 is a nonstructural protein first described 11 years ago [15]. This 79-90 amino acids long accessory protein is encoded by an alternative +1 reading frame on genomic segment 2, which also encodes the RNA polymerase basic protein 1 (PB1) and N40, an N-terminally truncated version of the PB1 protein which lacks transcriptase function [16]. PB1-F2 has been described to contribute to virulence of IAV. Expression of PB1-F2 from H1N1 viruses has been shown to enhance virus pathogenicity in mouse models of IAV infection [10], [12], [17]. A single N66S substitution present in the PB1-F2 of the 1918 pandemic strain and in some H5N1 HPAIV strains was shown to contribute to the severity of the disease caused by these highly virulent viruses in mice; this substitution has been proposed to represent one of the factors responsible of the high lethality of the 1918 virus in humans [11], [13]. However, PB1-F2 specific contribution to the pathogenic process, underlying molecular mechanism(s) and function(s) in the replication cycle of IAV remains matter of debate. Numerous studies have associated PB1-F2 with apoptosis induction in a cell type- and virus strain-specific manner [15], [18], with inflammation induction leading to secondary bacterial pneumonia [10], [17], [19], [20] and more recently with inhibition of interferon host response [21], [22], [23].

PB1-F2 involvement in cell death has been linked to its predominant mitochondrial localization, a mitochondrial targeting sequence (MTS) being located at its C-terminal part [24]. PB1-F2-induced apoptosis requires an infectious context to be revealed and is mediated by mitochondrial permeabilization, which is followed by a cytochrome c release [18]. Since 1947, seasonal H1N1 IAV isolates encode a truncated 57 aa-long form of PB1-F2 which lacks the C-terminal MTS and shows a diffuse cytoplasmic distribution [25]. Therefore, these recent H1N1-lineage viruses are likely to be affected in their capacity to induce cell death if mediated by mitochondrial interactions; since they usually caused milder symptoms than pre-40s H1N1 seasonal strains and also contemporary H3N2 seasonal strains which express a full length PB1-F2 protein, several authors have speculated on a relationship between IAV virulence in humans and their ability to induce apoptosis [12], [26]. However, PB1-F2 ability to cause cell death may be a property of some specific strains; whether this ability actually contributes to pathogenicity of epidemiologically relevant strains in humans remains a matter of debate [19].

Several works designed to understand the pathological processes mediated by PB1-F2 have indeed shown that its expression enhances the inflammatory response mounted by the host. PB1-F2 interferes with the host response leading to immunopathology by significantly increasing the leukocytes influx within the airways in mouse models based on recombinant viruses expressing various PB1-F2 proteins on the PR8 or WSN backbones or isogenic viruses with no PB1-F2 expression [10], [17], [19], [20], [23]. Expression of PB1-F2 was associated with an increased cellularity in the bronchoalveolar lavage fluids which largely consisted of macrophages and neutrophils, and also with an enhanced production of pro-inflammatory cytokines and chemokines.

In some cases, the underlying molecular mechanisms promoting the PB1-F2-mediated immunopathology appear to be linked to the inhibition of the early IFN response. Both the wild-type (WT) PB1-F2 protein of PR8 [22] and the N66S PB1-F2 allelic form found in the 1918 H1N1 pandemic virus and some H5N1 HPAIV strains [23] are able to delay the innate host response allowing a more efficient viral growth that finally generates a stronger infiltration of immune cells in the respiratory tract. The IFN inhibition mediated by PB1-F2 occurs by interfering with the RIG-I RNA sensory pathway at the level of the MAVS adaptor protein [22] and is more pronounced for N66S PB1-F2 [27]. Additionally, the dysregulation of host responses mediated by PB1-F2 appears to be strain specific. Studies made to restore the PB1-F2 open reading frame of the 2009 pandemic H1N1 IAV indicate that its expression has minimal effect on the modulation of the host immune response and virulence in mouse, ferret and swine animal models [28], [29], [30]. A similar observation was made in the context of the A/USSR/90/77 H1N1 seasonal strain that naturally expresses a truncated PB1-F2 [31]. Moreover, depending on the strain of the virus, PB1-F2 can exhibit pro- or non-inflammatory phenotypes: PB1-F2 from the 1968 H3N2 pandemic strain was shown to induce inflammation in mice lungs whereas those from more recent seasonal H3N2 viruses had lost this capacity to engender an inflammatory reaction [19]. Finally, the impact of PB1-F2 expression on virulence may also differ depending on the nature of the host: PB1-F2 contributes to virulence and systemic spreading of the A/Vietnam/1203/04 H5N1 HPAIV in ducks, but has only a minor impact on pathogenicity in mice; in contrast, the N66S polymorphism was shown to enhance pathogenicity, replication and neurotropism in mice but not in ducks [13].

As referred above, many, if not most studies have been performed on the PR8 or WSN backbone, so further investigations on PB1-F2 contribution to virulence in their native background are warranted. In this study, to assess comprehensively the impact of PB1-F2 expression on the mammalian host response to HPAIV infection, we compared the histological lesions induced in mice lungs by a fully reconstructed H5N1 HPAIV strain that was originally isolated in duck [A/duck/Niger/2090/2006, (Nig06)] with those mediated by its PB1-F2 depleted counterpart (ΔF2 Nig06). We then performed global transcriptomic analyses of the infected lungs at multiple time points post-infection (pi) with both viruses, and compared the data obtained. The analyses showed a transcriptional delay in the host response induction due to PB1-F2 expression within the airways. PB1-F2 modifies the immune response in a time dependent manner: it inhibits the early response of alveolar macrophages (2 days pi) and the activation of NK cells (4 days pi), and ultimately increases granulocytes recruitment and inflammation in the days preceding the death of the animals. Furthermore, we also showed that PB1-F2 expression is strongly associated to a gene signature suggesting that it promotes damage to the lung epithelium.

## Results

### Production and characterization of the recombinant WT and ΔF2 Nig06 viruses

To produce a PB1-F2 knock-out virus and its WT H5N1 HPAIV counterpart, we initially undertook the development and validation of a reverse genetics system for the production of a highly pathogenic H5N1 virus of avian origin. To that end, we first cloned all the sequences corresponding to the 8 genomic segments of the H5N1 A/duck/Niger/2090/2006 strain (further named Nig06) into the pRF483 bidirectional transcription plasmid. After co-transfection of the 8 plasmids in MDCK and HEK293T cells co-culture, we obtained a recombinant virus with similar phenotypic characteristics and high virulence in mice to those of the primary viral isolate cultivated in MDCK cells. Mouse 50% lethal dose (MLD50) ranged from 5–13 TCID50 in BALB/c mice to 22–34 TCID50 in C57BL/6 mice, death occurring between day 8 and day 12 after intranasal challenge with 10 MLD50 of virus. The virus knocked-out for PB1-F2 expression was similarly generated: the PB1-F2 in-frame ATG codons were modified by site-directed mutagenesis of the pRF483-PB1 plasmid without alteration of the encoded PB1 sequence. Genotype and phenotype of the PB1-F2 knock-out virus were confirmed by sequencing of the mutated PB1 segment. Both WT and ΔF2 Nig06 viruses were able to replicate efficiently and to generate cytopathic effects on MDCK. No difference in growth kinetics, yields of production and plaque size (not shown) between the two viruses could be observed in MDCK and murine pulmonary LA-4 cells.

**Figure 1:**
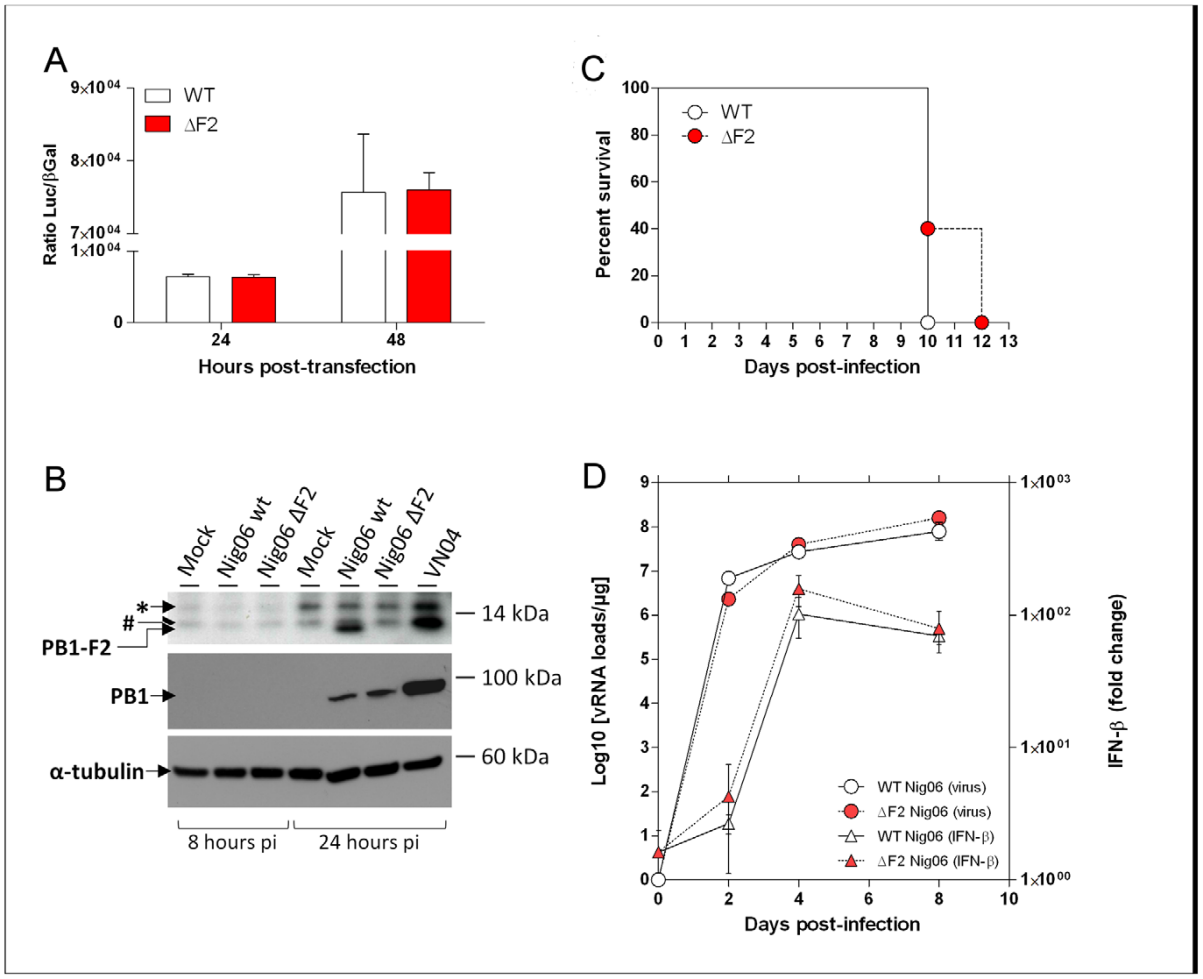
Characterization of the WT and ΔF2 Nig06 HPAIV. (A) Minigenome assays were used to compare polymerase activities of the WT and ΔF2 polymerase complex. Plasmids encoding the NP, PA, PB2 and PB1 or ΔF2-PB1 were co-transfected in 293T cells together with the reporter gene expressing plasmid pPolI-LUC allowing the quantification of polymerase activity. A plasmid encoding the pRSV-β-Gal was also cotransfected to control DNA uptake. Luciferase activity was measured in cell lysates 24 and 48 hours post-transfection. Data are expressed as the mean luciferase activity ± SEM of 3 replicates normalized to β-galactosidase activity. (B) Lysates from MDCK cells mock-infected, infected during 8h and 24h with the wt, ΔF2 or VN04 (A/Vietnam/1203/2004 (H5N1)) viruses (MOI = 5) were analyzed by Western blotting using anti PB1-F2, anti PB1 and anti α-tubulin antibodies. VN04 virus is used as a positive control for antibodies reactivity. Wt and VN04 viruses, but not ΔF2 virus, expressed PB1-F2 around 11 kDa as expected. Two non-specific bands also expressed in mock-infected cells are observed (*, #). The PB1 antibody is directed against the C-terminal part of PB1 and is able to recognize PB1 and N40. Wt and ΔF2 viruses expressed similar amount of PB1, indicating identical expression efficiency. No band corresponding to N40 was observed with every virus, possibly due to a weak expression of N40 which cannot be detected by the PB1 antibody. Alpha-tubulin is used as loading control (C) Lethality induced by WT and ΔF2 Nig06 viruses in C57Bl/6 mice. Mice received intranasally (n = 5) 200 TCID50 of WT or ΔF2 Nig06 virus and were observed every two days for mortality. (D) Time course of M vRNA and IFN-β mRNA expression in the airways of infected mice. Mice were infected with 200 TCID50 of WT or ΔF2 viruses and were euthanized at 2, 4 and 8 days pi. After extraction, lung total RNAs were reverse transcribed and used to quantify viral load and IFN-β transcription. Viral loads were evaluated as M vRNA copies and normalized to the amount of RNA engaged in the reaction; IFN-β mRNA levels were normalized to β-actin mRNA levels and presented as fold increase relative to mock-treated mice.

To further investigate whether Nig06 PB1-F2 may modulate the viral RNA-polymerase activity as shown for some other virus strains [26], [32], we used a luciferase minigenome replication/transcription assay to compare the enzymatic activities of the WT and ΔF2 polymerase complex. To this end, plasmids encoding the Nig06 PA, PB2, NP proteins and the luciferase reporter gene were cotransfected in 293T cells with the plasmid encoding the PB1 wild-type segment or the PB1 segment deleted for the PB1-F2 ORF. No significant difference in the reporter gene expression was observed at any time post-transfection, whatever the PB1 segment used (Fig. 1A). Therefore, Nig06 PB1-F2 does not modulate the viral RNA-polymerase activity in cells of mammalian origin. We then aimed to determine whether the mutations introduced to invalidate PB1-F2 had an effect on the expression of N40, a N-terminally truncated form of PB1 as previously published [16]. Western blot analysis confirmed that the ΔF2 Nig06 virus was not able to express PB1-F2 (Fig. 1B). We next compared the expression of PB1 and N40 by the WT and ΔF2 Nig06 virus. No difference was evidenced in the expression profile of PB1 and N40 (Fig. 1B). Densitometric measurements of PB1 levels standardized to α-tubulin levels on independent experiments confirmed that mutations introduced had no effect on PB1 and N40 expressions (data not shown), allowing us to exclude the involvement of N40 in host-response differences processes. To further characterize the effect of PB1-F2 on virus growth and virulence *in vivo*, we first determined the MLD50 of both WT and ΔF2 Nig06 viruses after intranasal inoculation to C57BL/6 mice. Their MLD50 were calculated to be around 10^1.5^ and 10^0.9^ TCID50, respectively, a small difference that we considered not significant. Moreover, kinetics of weight loss, signs of either sickness or death did not notably differ in the groups infected with 200 and 40 TCID50 of either virus. Survival curves of the animals infected at the 200 TCID50 dose are represented in Fig. 1C. Mortality appeared between days 9 and 12 pi and no statistically significant difference in the mean time to death was detected between the 2 groups of mice.

Recent studies showed that PB1-F2 exacerbates IFN-β expression in mouse infected by the WSN low pathogenic viral strain [17], and conversely display interferon antagonism for some other strains [22], [23]. Therefore, in a separate experiment, we infected C57BL/6 mice intranasally using 200 TCID50 of each virus, the minimal dose that induced 100% mortality for both viruses in the above preliminary dose-range experiments. Three mice of each group were euthanized at 2, 4 and 8 days pi and lungs were collected to evaluate viral loads and IFN-β induction (Fig. 1D). Viral RNA quantification in lungs of infected mice showed fast and efficient replication of both viruses at 2 and 4 days pi, and, remarkably, the viral loads were not reduced at later time points but still increased at 8 days pi. We also observed an increase in IFN-β mRNA levels: IFN-β transcription was induced as early as 2 days pi, peaked at 4 days pi and then slightly decreased at 8 days pi. Viral loads and IFN-β curves were statistically equivalent between the 2 viruses. Genomic stability of both viruses was confirmed by nucleotide sequencing of the segment #2 at the different time points.

Collectively, these data show that Nig06 PB1-F2 has no impact on the replication of the virus *in vitro* and *in vivo* and does not modulate the induction of the antiviral mediator IFN-β in mice.

### Pathogenesis associated to PB1-F2 from Nig06

**Figure 2:**
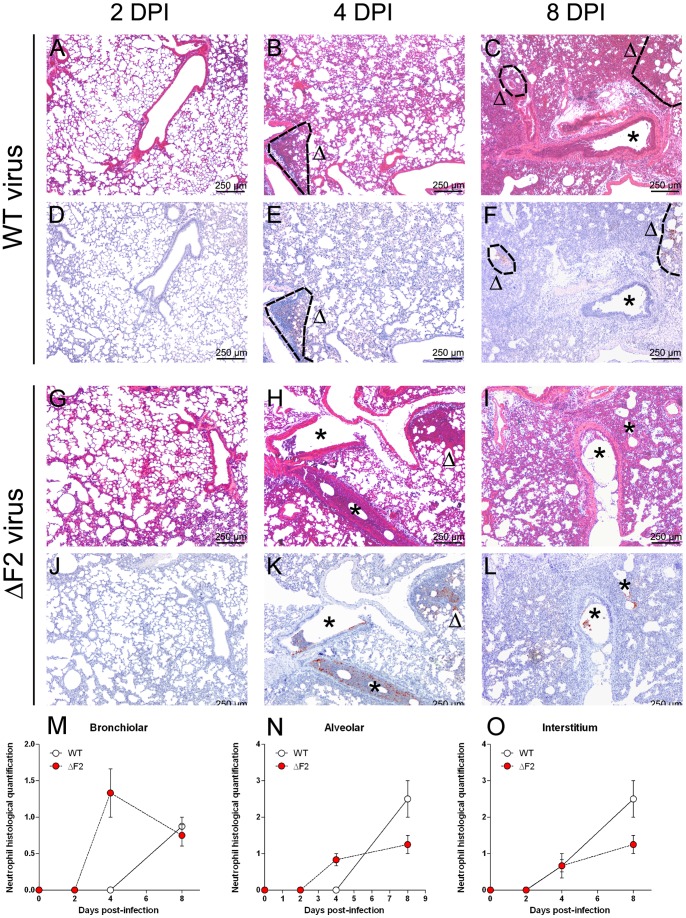
Confrontation of pulmonary histological lesions after intranasal inoculation of wild type and Δ F2 viruses. Groups of 3 mice were infected intranasally using 200 TCID50 of WT or ΔF2 Nig06 virus. Mice were euthanized at 2, 4 and 8 days pi (dpi) and lungs were processed to allow histopathological examination. WT virus. (A, D) 2 dpi: no histological lesion. (B) 4 dpi: inflammatory infiltrate centered on alveoli/interstitial tissue, in the periphery of a bronchiole, (E) presence of infected cells in the inflammatory infiltrate. (C) 8 dpi: more severe inflammation in alveoli/insterstitial tissue associated with (F) a high number of infected cells. ΔF2 virus. (G, J) 2 dpi: no histological lesion. (H) 4 dpi: inflammatory infiltrates centered on bronchi/bronchioles, (K) Infected cells are identified in the inflammatory infiltrates. (I) 8 dpi: inflammatory lesions persist in the bronchi/bronchioles but are associated with more severe lesions in the alveoli/interstitium. (L) Infected cells could be detected in inflammatory lesions. *(A-C, G-I: HE staining; D-F, J-L: immunohistochemistry with anti-H5N1 antinbodies;* Δ*: alveoli/interstitium, asterisk: bronchi/bronchioles)*. Time-course of neutrophil infiltration. Neutrophils were quantified within bronchi/bronchioles (M), alveoli (N) and interstitium (O) compartments by histological observation. Results presented are the mean graded scores of 3 replicate animals ± SEM.

To further characterize the effect of PB1-F2 *in vivo*, we performed thorough histological investigations of the lungs of C57BL/6 mice infected with 200 TCID50 of WT or ΔF2 Nig06 virus. The aim of the histopathological analysis was to decipher the consequences of PB1-F2 expression during C57BL/6 mouse infection at the tissue scale. Many lesion patterns were similar between WT and ΔF2 virus infection (Fig. 2). Both indeed displayed: (i) an absence of lesion and apparent infected cells 2 days pi (Fig. 2A, D, G, J), (ii) necrosis and infiltration of neutrophils, macrophages and lymphocytes 4 days pi (Fig. 2B, E, H, K), (iii) an increase in the severity of inflammatory lesions at day 8 pi (Fig. 2C, F, I, L) and (iv) the presence of viral antigen in epithelial cells, macrophages and cell debris 2 and 4 days pi. However, many differences were identified, suggesting that PB1-F2 expression has a significant impact in the pulmonary tissue during infection. These differences were: (i) the early targeting of bronchi/bronchioles by the ΔF2 virus, 4 days pi, characterized by the presence of the virus and a much higher infiltration of neutrophils (Fig. 2H, K and M), (ii) a higher number of infected cells in the bronchi/bronchioles of ΔF2-infected mice at day 8, (iii) a more severe inflammation in WT-infected mice at day 8 pi (Fig. 2C, F) associated with (iv) a lower number of infected cells and more severe lesions in the alveoli and interstitial tissues (Fig. 2C, F), where the recruitment of neutrophils was significantly more important (Fig. 2N, O).

Collectively, these data suggest that PB1-F2 may delay the inflammatory response in the upper airways of the infected host and be responsible of extensive alveolar damages at late time of infection.

### Kinetic analysis of the host transcriptional response to WT and ΔF2 Nig06 viruses

**Figure 3:**
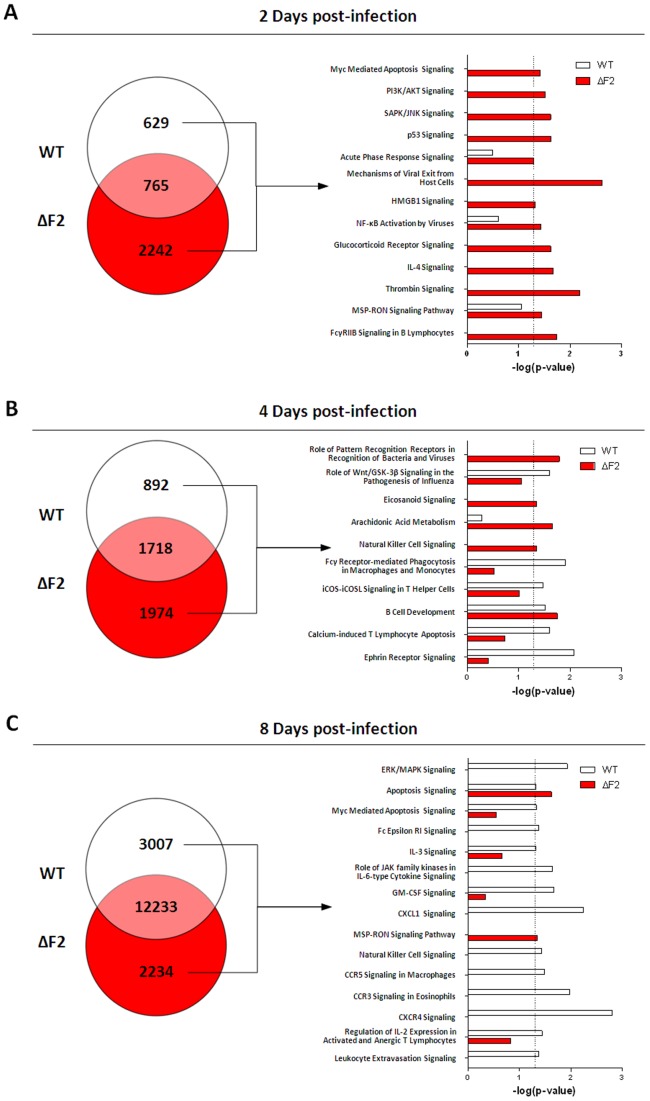
Summary of genes regulated during infection by the WT Nig06 or by the ΔF2 Nig06. Total RNA from lungs of mice infected by the WT (white) and ΔF2 (red) viruses were extracted at 2 days pi (A), 4 days pi (B) and 8 days pi (C), and then processed to be analyzed on pangenomic mouse microarrays. Venn diagrams show the distribution of differentially regulated genes (*i.e.* infected *vs.* mock-infected) during infection with WT or ΔF2 Nig06. The data presented were compiled from replicate RNA samples prepared from 3 to 4 individual mice and analyzed on independent microarrays. Functional categories associated to the differentially expressed genes were identified using IPA and represented as histograms. These results are presented as the negative logarithm of significance, which corresponds to the likelihood of the genes of a given pathway being found together as a result of chance, as determined by Fisher's exact test.

To further investigate the impact of PB1-F2 expression on the pathogenic process induced by the Nig06 virus, we compared the host gene expression levels in the lungs of mice infected with the WT and the ΔF2 virus. Total RNA was extracted from the lungs of C57BL/6 mice infected with 200 TCID50 of either WT or ΔF2 Nig06 virus and euthanized at 2, 4 and 8 days pi. Expression microarray analysis was performed by comparing every samples prepared from the lungs of WT or ΔF2 Nig06-infected mice to samples prepared from lungs of mock-infected mice. Differentially expressed genes during either infections were defined as having a 20% fold change in expression levels compared to mock-infected samples (p value<0.05). The lists of genes regulated by the two viruses from combined replicate experiments were then compared using Venn diagrams (Fig. 3). Genes specific for each virus were used to characterize the functional consequences of PB1-F2 expression. We used Ingenuity Pathway Analysis (IPA) software to identify canonical pathways associated to the differentially expressed genes. The significance of each identified pathways are represented in the histograms of Fig. 3.

At day 2 pi, global gene expression patterns indicate that the ΔF2 virus regulated a more important number of genes than the WT virus (3007 *vs.* 1394; Fig. 3A). This is an unexpected result given that in our previous mouse model, using the low pathogenic WSN H1N1 virus [17], the WT virus induced a much more intense response than the ΔF2 homologous virus. The most remarkable insight is that only ΔF2-regulated groups of genes reached statistical significance (i.e. p<0.05, dashed line), indicating that the associated canonical pathways are significantly regulated during infection by the ΔF2 virus but not during infection by the WT virus. Among the most significantly regulated pathways, 3 of them are tightly associated to macrophage activation: “Acute Phase Response Signaling”, “HMGB1 Signaling” and “MSP-RON Signaling”. Another major information given by the transcriptome analysis at day 2 pi is the importance of PI3K/AKT pathway and to a lesser extent of SAPK/JNK signaling during infection with the ΔF2 virus. The PI3K/AKT pathway appears to be at a central position of the host response process since it is implicated in almost all the other 12 pathways represented in Fig. 3A. The data collected at day 2 pi suggest that PB1-F2 expressed from WT Nig06 IAV disturbs directly or indirectly the alveolar macrophages function and may therefore delay the mounting of an efficient antiviral defense by the host.

Four days pi, differences in the intensity of the host response tend to decrease between the 2 viruses. Lungs of mice infected with the ΔF2 virus express more genes dedicated to the recognition of pathogens and to the metabolism of lipids including genes responsible for production of pro-inflammatory lipids (Eicosanoids). “Natural Killer Cell Signaling” pathway is also highly regulated in the absence of PB1-F2 expression. NK cells are component of the innate immune system able to provide a rapid response to virus-infected cells [33]. Gene expression patterns specifically observed with the WT virus at day 4 pi clearly associate the expression of PB1-F2 with changes in the behavior of immune cells like monocytes/macrophages and lymphocytes. PB1-F2 expression appears to modify the phagocytosis capacity of macrophages and monocytes. PB1-F2 also contributes to T-cells activation by increasing the ICOS-mediated signaling. This activation is associated with an increase of the Ca^2+^-mediated apoptosis of T-cells due to the upregulation of genes implicated in the release of Ca^2+^ from intracellular stores (IP3R voltage-gated calcium channel and SERCA sarcoplasmic reticulum calcium pump). Ephrin receptor signaling is also associated with PB1-F2 expression; ephrin receptors are large group of receptor tyrosine kinases with capacity to influence cell behavior like cell migration.

At day 8 pi, the transcriptome of infected mice reveals a major gene regulation: more than 15,000 genes are regulated in the lungs of WT-infected mice. This coincides with the potent immune cell recruitment within the alveolar compartment 2 days before the death of the animals. Interestingly, at this time point, WT virus infection significantly increases the expression of genes involved in leukocyte chemoattraction and especially in the recruitment and activation of polymorphonuclear granulocytes: IL3 signaling, GM-CSF signaling, CXCL1 signaling, CCR3 signaling and CXCR4 signaling (Fig. 3C). This PB1-F2-dependant gene signature of the lung transcriptome concurs with the histological description of the granulocyte infiltration which characterizes infection by the WT virus at day 8 pi. We also observed a regulation of NK cell signaling in WT-infected mice but not in ΔF2-infected mice. This suggests that PB1-F2 delays the activation of NK cell activation given that this pathway is activated earlier in the infection (day 4 pi) with the ΔF2 virus, as described above.

As a whole, our kinetic analysis of the host transcriptional response indicates that PB1-F2 strongly interferes with the immune response mounted by the host throughout the pathological process.

### PB1-F2 from Nig06 delays immune gene regulation

**Figure 4:**
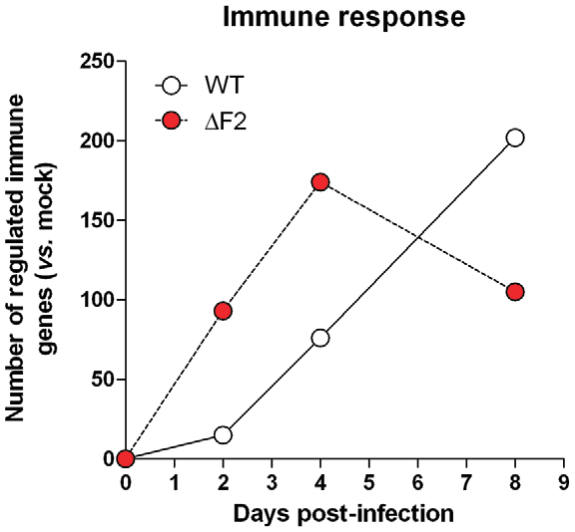
Global transcriptional response of genes indexed as “immune genes”. The number of immune genes regulated during the time course of infection by the WT (white) and the ΔF2 (red) viruses were compared. Genes were filtered with the term “immune response” (Biological Process) using the Gene Ontology Database: accession GO:0006955. Gene expressions in lung tissues of infected mice were compared to those of mock-infected mice and considered as regulated if they reached 20% expression fold change (p-value<0.05).

Pathways relevant to the immune response are prevalent in the transcriptomic signature associated to PB1-F2 expression during infection. In order to evaluate the specific regulation of immune genes during infection by the 2 viruses, we filtered all the regulated immune genes of the transcriptomic analysis with the term “immune response“ using the Gene Ontology Database (GO:0006955). As shown in Fig 4, the global pattern of immune genes regulated by the WT Nig06 is particularly low at days 2 and 4 in comparison to the ΔF2 Nig06 infection. However, it displays a continuous increasing profile over the time of infection. By contrast, the number of immune genes regulated during the infection by the PB1-F2 deleted virus decreases at day 8 pi. These data closely match to the histological description of the airways inflammation and also to the severity of lung pathology observed during the infection.

### PB1-F2 from Nig06 promotes tissue damage and delays alveolar macrophages activation

**Figure 5:**
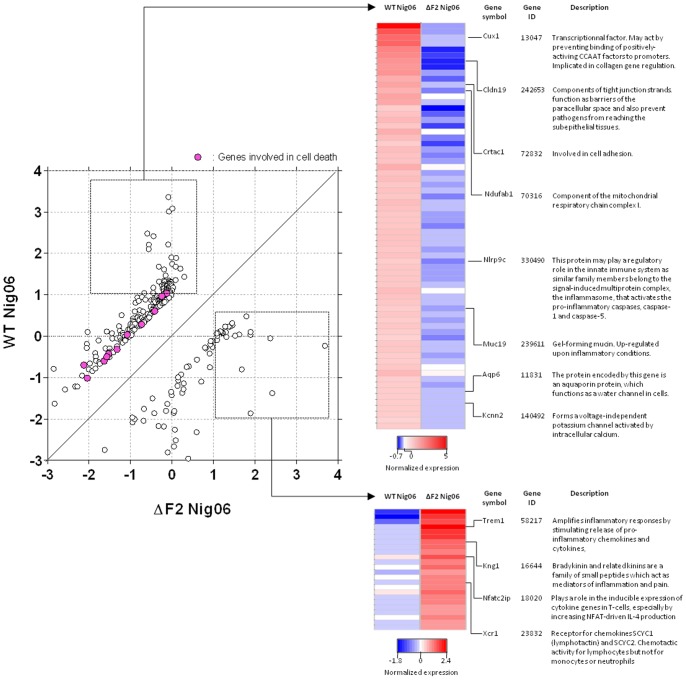
Expression profiles of genes affected by the WT Nig06 and the ΔF2 Nig06 in lungs of mice at 2 days pi. Left part of the figure represents a scatterplot comparing normalized expression values in WT-infected and ΔF2-infected mice of a subset of highly differentially expressed genes. A difference of 1 between the normalized expression values was defined as the cutoff for a differentially regulated gene. Several genes annotated by “Gene Ontology” as implicated in cell death are highlighted in purple. Genes induced in each of specific conditions were then selected and represented in the right part of the figure as a heat map. For each gene, the data presented are the average expression levels at day 2 pi and were calculated from replicate RNA samples prepared from 3 individual mice and analyzed on independent microarrays. Genes shown in red are up-regulated and those shown in blue are down-regulated in infected mice compared to mock-infected mice. Data are expressed in Log base 2 ratio. Examples of genes with details on the cellular functions are indicated.

As shown in Fig. 3A, no pathways were significantly regulated in the WT Nig06-infected mice at 2 days pi. To get insight into the critical events occurring at this time point and to identify functions that could be non-indexed in IPA software, we did a closer inspection of the genes preferentially regulated by the two viruses at day 2 pi. The scatterplot depicted in Fig. 5 illustrates the expression of the most differentially regulated genes in the 2 types of infection. Genes implicated in cell death (highlighted in purple) are regrouped in the cluster of genes specifically regulated in mice infected by the WT virus, indicative of an increase in apoptosis in the presence of PB1-F2. These results correlate well with the disturbance of alveolar macrophages function evidenced above, and also the initial observation that PB1-F2 may promote apoptosis in certain cell types, especially monocytes [15]. Although the role of apoptosis during influenza virus infection is controversial, PB1-F2 has therefore been proposed to disable virus-infected alveolar macrophage, thus helping the virus to evade detection by the immune system [34], [35].

We then focused on the genes highly induced in only one condition (*i.e.* specific for a virus). Genes displaying a normalized expression level >1 in one condition and <0.5 in the other condition were selected. These genes are represented in the two heat maps of the right part of Fig. 5. Several genes involved in the architecture of the epithelium and in the modification of the mucus properties were found in the cluster of WT Nig06-regulated genes: Cux1, Cldn19, Crtac1, Muc19, Aqp6, Kcnn2. Cux1, is one of the most differentially regulated gene between the WT-infected and the ΔF2-infected mice (8,6 and 0,8 fold change vs. mock respectively). It encodes a transcriptional factor involved in tissue homeostasis in several organs. Abnormal CUX1 expression may for instance lead to aberrant expression of type I collagen [36]. This up-regulation of CUX1 is of particular interest since several other genes in the cluster possess one or multiple CUX1 binding sites in their promoter (not shown). CUX1 could therefore occupy a central regulatory position in this PB1-F2 specific cluster of genes.

By contrast, the pool of genes induced during ΔF2 Nig06 infection is mainly involved in the mounting of an immune response: Trem1, Kng1, Nfatc2ip and Xcr1 are particularly implicated in mediation of inflammation, cytokine induction and leukocyte chemoattraction [37].

Collectively these gene signatures suggest that PB1-F2 promotes damage to the airway epithelium during Nig06 virus infection and delays resident immune cells activation.

### PB1-F2 from Nig06 transiently impairs NK cell activation

**Figure 6:**
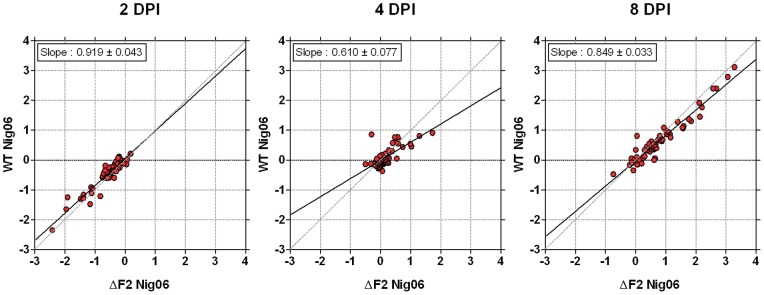
Expression profiles of NK cell-specific genes. The 3 scatterplots compare normalized expression levels of NK cell-specific genes during infection with WT Nig06 (y axis) and ΔF2 Nig06 (x axis) viruses at different times pi: 2 days pi (left panel), 4 days pi (middle panel) and 8 days pi (right panel). Data are expressed in Log base 2 ratio. The linear regression curves (black lines) and the slope of the curves are represented for each scatterplot.

The pathway analysis indicated a differential activation of NK cells at day 4 pi (Fig. 3B). To confirm that expression of NK cell-specific genes were altered at this time-point in presence of PB1-F2, we focused our analysis on the expression of 58 genes involved in NK-cell activities (lectin-like receptor involved in “natural killer cell activation”: GO:0030101). The expression patterns of these genes are represented in Figure 6. The 3 scatterplots compare the dynamic expression profiles of the selected 58 NK cell-specific genes in the lungs during infection by the 2 viruses. Interestingly, the magnitude of expression significantly augmented over the time after infection, suggesting an increasing involvement of NK-cells in the host response during the course of infection. For example, the lectin-like transmembrane receptor Klra2 (also known as Ly49b, Gene ID: 16633) displays a fold change of expression (WT Nig06 *vs.* mock) of 1.2 at day 2 pi, 2.1 at day 4 pi and 5.7 at day 8 pi. Linear regression analysis performed on expression levels in WT-infected mice versus expression levels in ΔF2-infected mice indicates that the 58 NK cell-specific genes displayed a higher level of expression at 4 days pi with the ΔF2 Nig06 virus (slope  = 0.61; Fig. 6 middle panel). The individual differences observed are relatively modest, for example the fold change of expression (*vs.* mock) of the lectin-like receptor Klra12 (also known as Ly49L, Gene ID: 16630) is 1.75 during WT Nig06 virus infection and 2.46 during the ΔF2 Nig06 virus infection. However, the global expression of the entire set of NK cell-specific genes displays a higher level of expression during the ΔF2 Nig06 virus infection, suggesting that PB1-F2 may impact the NK cells functions during infection with Nig06. It is noteworthy that this effect is transient since it is only observed at 4 days pi, NK cell genes expression returning to almost identical levels at 8 days pi (Fig. 6 right panel). Altogether, our data support the hypothesis that expression of PB1-F2 delays the activation or recruitment of NK cells in the infected lungs.

### PB1-F2 from Nig06 increases recruitment of granulocytes at day 8 pi

**Figure 7:**
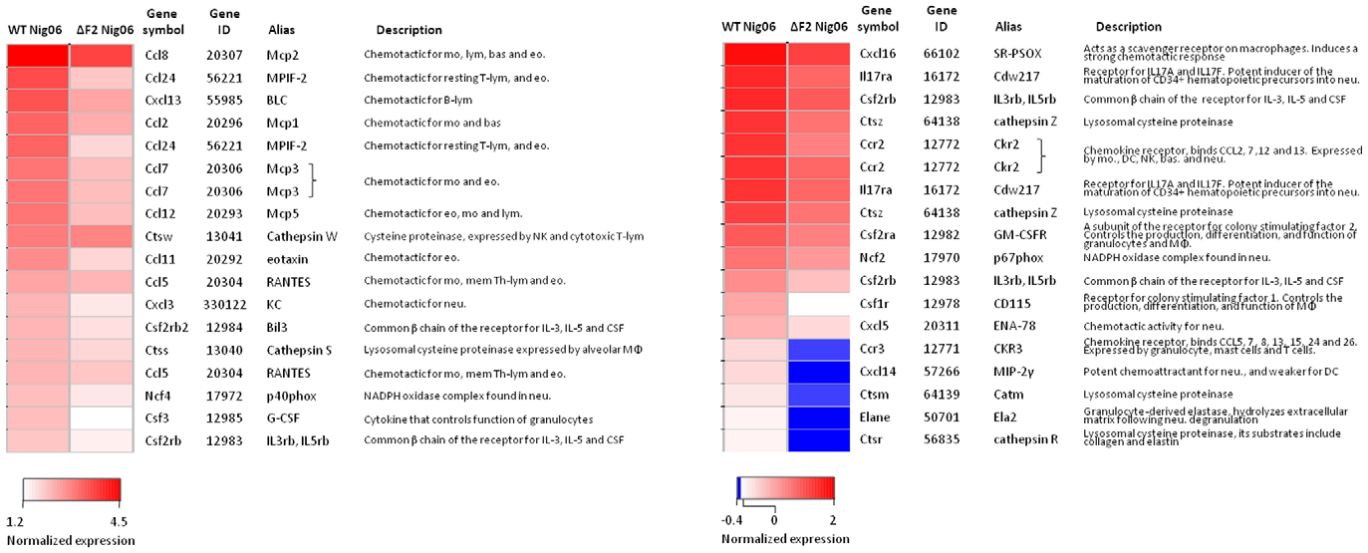
Heat map of selected genes related to granulocytes functions and chemoattraction. For each gene, the data presented are the average expression levels at day 8 pi and were calculated from replicate RNA samples prepared from 4 individual mice and analyzed on independent microarrays. Genes shown in red are up-regulated and those shown in blue are down-regulated in infected mice compared to mock-infected mice. Data are expressed in Log base 2 ratio. Abbreviation: mo, monocyte; lym, lymphocyte; bas, basophil; eo, eosinophil; NK, natural killer; mem Th-lym; memory T-helper lymphocyte; neu, neutrophil; MФ, macrophage; DC, dendritic cell.

The histological analysis of the lungs (Fig. 2) and the pathway analysis (Fig. 3C) pointed out a prominent neutrophilic recruitment in the lungs of mice, 8 days after infection with the WT Nig06 virus. To go further into detail, we filtered genes important in the chemoattraction and in the function of granulocytes, and represented their expression levels at day 8 pi in the heat map of Fig. 7. These genes display a more robust transcriptional regulation in WT Nig06-infected mice in comparison to ΔF2 Nig06-infected mice. Among the chemokine genes represented in the heat map of Fig. 7, CCL24 displays the most important difference of expression between the WT and the ΔF2 Nig06 viruses (two probes): 13.7 and 9.7 fold increase *vs.* mock for the WT Nig06 and 4.6 and 3.7 fold increase *vs.* mock for the ΔF2 Nig06. CCL24 (also known as eotaxin-2 in humans) interacts with the CCR3 chemokine receptor and induce chemotaxis of granulocytes [38]. Interestingly, the CCR3 gene is also differentially expressed between the 2 infection conditions. In addition to granulocyte chemotactic factor genes, other genes were also more expressed in WT-infected mice including genes encoding a cytokine that controls the function of granulocytes: CSF3 (also known as GCSF), and genes encoding specific components of neutrophils: Ncf2 and 4 (neutrophil cytosolic factor 2 and 4), Elane (Elastase) and Cathepsin G (not shown). Altogether, these data underline the proinflammatory role of PB1-F2 and its capacity to induce the recruitment of neutrophils into the airways during the days preceding the death of the infected host.

## Discussion

Various factors are thought to be involved in the pathogenesis of H5N1 HPAIV; among them, the multibasic cleavage site of the hemagglutinin [9], [39] and the enhanced ability of NS1 to suppress innate immunity have been very well described [40], [41]. PB1-F2 is also implicated in highly pathogenic IAV virulence [11], [13], [14], [19], [23]. In this study, we investigated the effect of PB1-F2 expression in lungs of mice during infection by a HPAIV. For this purpose, we took advantage of the reverse genetic tool to produce and characterize a HPAIV unable to express the PB1-F2 protein. As it was observed that PB1-F2 interferes with the host response in a manner dependant of the nature of the host, the strain of the virus and its sequence polymorphism [11], [13], [15], [19], [26], it was important to use a virus isolated from the field and able to infect avian and mammal hosts. Here we used the strain A/duck/Niger/2090/2006 (H5N1) which has all the characteristics of a HPAIV and which is highly virulent in the mouse model of infection but does not harbor the N66S single point mutation on PB1-F2. The presence of a serine at position 66 has indeed been associated with increased virulence of IAV [11] but is present within only 5.1% of the 954 PB1-F2 sequences from H5N1 strains referenced in the NCBI Influenza Virus Resource Database [42]. The PB1-F2 from Nig06 is therefore representative of most of the PB1-F2 from HPAIV.

In mouse models, HPAIV usually induce fast and high mortality with very low dose inocula, for example MLD50 of A/Vietnam/1203/04 virus is 3.2 PFU in C57Bl/6 and death happens as early as 5 days pi [13]. In our study, MLD50 of the Nig06 is 36 TCID

50 (∼25 PFU) but remarkably, the mortality arises very late: between 9 and 12 days pi. This particularity gave us the opportunity to study several critical time points during the infection: 2 days pi (high replication and beginning of the immune response), 4 days pi (damage of the epithelia) and 8 days pi (massive leukocyte infiltration of the alveolar lumen, period preceding the mice death). Of note, the deletion of PB1-F2 does not influence the outcome of the infection (Fig. 1C). We hypothesized that the lack of difference in mouse lethality must be due to the extreme virulence of the Nig06 strain. Mx+/+ mice should be used to highlight variations in the lethality mediated by PB1-F2 expression as previously described by Schmolke *et* *al.* [13].

**Figure 8:**
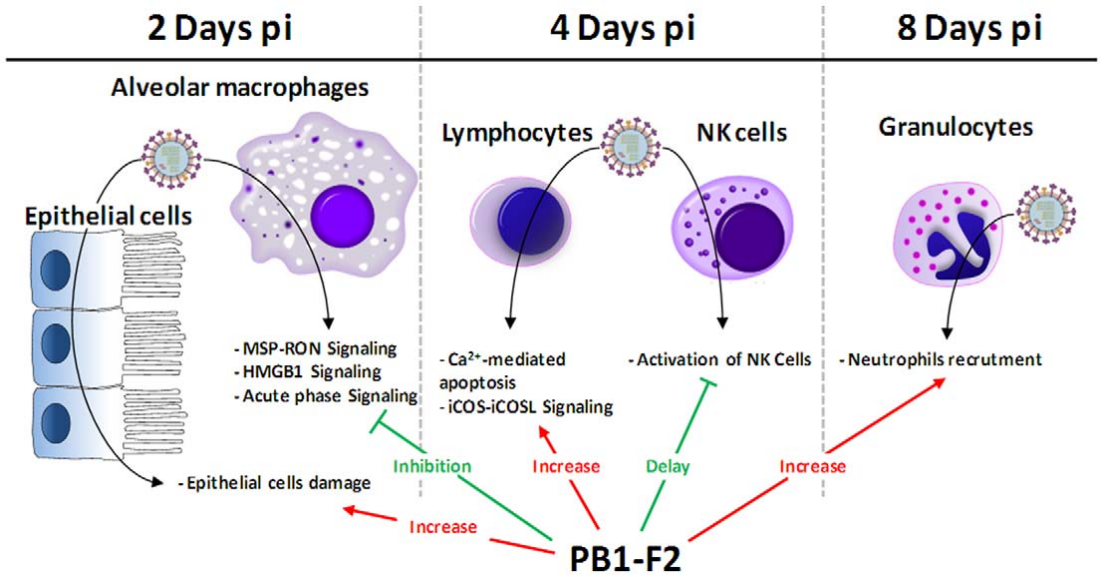
Summary of the principal host responses affected by PB1-F2 during infection of mice by Nig06 IAV. The dynamics of the host response associated to PB1-F2 suggests multiple effects and multiple target cells depending of the stage of the disease and depending on the location of the infection within the airways. At day 2 pi PB1-F2 lead to inhibition of cellular pathways implicated in macrophages activation. PB1-F2 is also associated to a gene signature suggesting that it promotes damage to the epithelial structure. At day 4 pi, the gene profile specific for PB1-F2 reveals a delay in the NK cells recruitment and function and an increase in Ca2+-mediated lymphocyte apoptosis. At day 8 pi, the expression of PB1-F2 is clearly associated with increased transcription of genes encoding chemokines and cytokines implicated in the recruitment of granulocytes.

We used functional genomics to analyze the host response associated to PB1-F2 from Nig06. Fig. 8 summarizes the main pathways and cell types affected by PB1-F2 expression during the kinetic of infection. Regarding the chronology of the disease, PB1-F2 expression contributes to pathogenesis at multiple levels depending on the time pi: epithelial cells damage, macrophages dysfunction, lymphocytes and NK cells dysfunction and granulocytes recruitment. These effects match with the progression of the pathology within the lower respiratory tract: from the trachea and bronchi to the lungs (alveolar compartment).

The gene signature linked to the expression of PB1-F2 from Nig06 was fundamentally different from the signature associated to PB1-F2 from WSN/33 IAV that we previously described [17]. PB1-F2 from Nig06 appears to delay the mounting of the immune response at early time points, whereas PB1-F2 from WSN/33 increases the intensity of the host response as soon as 2 days after infection. These contradictory effects may be linked to the kinetic of the pathogenesis process which is very slow in the case of the Nig06 in comparison to the WSN/33 (mean time of death: 10 days *vs.* 5 respectively). Conenello and collaborators have previously described an early inhibitory effect of PB1-F2 on the IFN host response [23]. In their model, the virus used was a reassortant with the A/WSN/1933 backbone and the PB1 segment from the A/Hong Kong/156/1997(H5N1). The PB1-F2 expressed by this virus arbor the N66S polymorphism responsible for an enhanced virulence. The data obtained by Conenello and collaborators strongly suggested that the N66S PB1-F2 inhibited the early IFN response and induced an uncontrolled growth of the virus that ultimately triggered a hyper inflammation of the lungs. In our HPAIV model with a PB1-F2 allelic form without the N66S substitution, the host response appears to be regulated in a different way since the IFN responses are strictly identical in WT and ΔF2 Nig06 infections. Such differences in the pathological mechanisms mediated by H1N1 and H5N1 highly virulent IAV have previously been described [43]. Interestingly, the PB1-F2 sequence differences between H1N1 and H5N1 virus strains are significant (Figure S1) and could explain such variations in the virulence process mediated by PB1-F2: PB1-F2 from WSN33 and Nig06 share only 63% identity.

In our Nig06 mouse model, at day 2 pi the main pathways inhibited by the expression of PB1-F2 are strongly associated with macrophage functions: acute phase response signaling, HMGB1 signaling and MSP-RON signaling pathways (Fig. 8). The acute phase response is a fast inflammatory response that protects against tissue injury using non-specific defense mechanisms. HMGB1 is an alarmin, an endogenous molecule able to activate the innate immune system [44]. HMGB1 can be passively released during necrosis or actively secreted by immune cells such as macrophages, and then signal through the receptor for advanced glycation end-products (RAGE), a multi-ligand receptor of the immunoglobulin superfamily, expressed on monocytes and macrophages [44].

PB1-F2 expression also inhibits the MSP-RON pathway. Macrophage Stimulating Protein (MSP) is secreted by the liver into the blood as pro-MSP. Tissue injury leads to pro-MSP cleavage and then binding to the transmembrane receptor kinase RON expressed on macrophages. This binding results in PI3K activity and production of cytokines such as IL-12. These cytokines in turn activate NK cells [45]. This is of first importance since we identified at day 4 pi a transient deficit in genes implicated in NK cells functions within the airways of WT Nig06-infected mice (Fig. 8). This deficit is probably the consequence of the macrophage pathways inhibition observed at day 2 pi and particularly the MSP-RON pathway. However we cannot exclude a direct effect of PB1-F2 on the NK cells.

Interestingly, when focusing on the genes preferentially expressed during the WT Nig06 infection at day 2 pi, we observed genes implicated in cell death and genes related to epithelial tissue repairing and homeostasis, suggesting that PB1-F2 from Nig06 promotes apoptosis and damage of the respiratory tract. This tissue injury signature is probably in relation with the capacity of PB1-F2 to destabilize membranes by forming pores [46], [47], [48]. The genes involved in cell death processes are most likely expressed by the alveolar macrophages since epithelial cells were never evidenced as sensitive to PB1-F2 in term of apoptosis [20] in contrast to immune cells [15]. It is therefore tempting to speculate that alveolar macrophages depletion through apoptosis could be the explanation of the immune response delay provoked by PB1-F2.

Another striking difference between the WT and the ΔF2 Nig06 infection at day 4 pi is the impact of PB1-F2 on T lymphocytes. The expression of PB1-F2 during the infection exacerbates the T cell activation through the ICOS signaling pathway. Signaling through ICOS enhances T cell proliferation, secretion of cytokines, up-regulation of cell surface molecules and also controls of the selective entry of Th1 cells into inflamed peripheral tissue [49]. Release of Ca^2+^ ions has a central position during activation of this pathway and is also implicated in apoptosis of T lymphocytes which is more important in presence of PB1-F2. Indeed, according to the pathway analysis (Fig. 3B), the T lymphocytes are more subjected to Ca^2+^-mediated apoptosis when mice are infected by the WT Nig06 virus (Fig. 8). It is important to mention that during IAV infections, infiltrating virus-specific T_eff_ cells contribute to inflammatory control through production of several pro- and anti-inflammatory mediators [50]. The induction of apoptosis in such immune cells could disrupt the inflammatory regulation and explain the differences observed in the magnitude of inflammation developed in presence or absence of PB1-F2 at day 8 pi. PB1-F2 was identified because it is presented by the MHC-I complex and recognized by epitope-specific CD8^+^ T cell populations [15]. While PB1-F2 is a target of the adaptive immune system, it is interesting to observe that PB1-F2 seems to increase the apoptosis of cells implicated in this process.

Our mouse model of HPAIV infection gave us the opportunity to study the late time points that are preceding the death of the animals. At day 8 pi, our analysis revealed an increase in the expression of genes implicated in the recruitment and activation of granulocytes (Fig. 8). This increase is significantly more important when PB1-F2 is expressed during the infection, suggesting that, at this time point, PB1-F2 from Nig06 exerts a pro-inflammatory effect and enhances the inflammation mediated by the HPAIV infection. In addition, the transcriptomic data related to the granulocyte recruitment associated to PB1-F2 are strongly supported by the histological examination (Fig. 2A). Why PB1-F2 delays the early response and amplifies the late response remains unknown, however this could be due to an increase in PB1-F2 concentration associated to a different location of the protein: at day 8 pi, the virus has indeed reached the alveolar compartment (Fig. 2B) and still replicate efficiently (Fig. 1D). Moreover, the time course of the infection exposes successively PB1-F2 to different types of immune cells recruited within the lung; consequently, PB1-F2 could play multiple roles depending on the stage of the infection. The increase in the granulocytes recruitment mediated by PB1-F2 appears to be a typical signature of PB1-F2 expression by various pathogenic IAV since it has been previously described in mice infected by A/WSN/1933, recombinant A/PR8/1934 viruses expressing the PB1-F2 of the 1918 pandemic strain or with A/PR8/1934 reassortants encoding PB1 segments from H3N2 or H5N1 viruses [10], [17], [19], [23]. The augmentation of granulocyte cellularity within the lung is concomitant with the onset of clinical symptoms of the disease and ultimately with death of the mice.

Collectively, our results describe the contribution of PB1-F2 in the pathologic process mediated by HPAIV infection of mammals. PB1-F2 interferes with the normal development of the immune response by first delaying the mounting of the immune response and then by amplifying the inflammatory response. These findings may help to elucidate the role of PB1-F2 in the influenza virus cycle and associated pathogenesis.

## Materials and Methods

### Ethics statement

This study was carried out in accordance with Pasteur Institute and INRA guidelines in compliance with European animal welfare regulation (http:// ec.europa.eu/ environment/chemicals/ lab_animals/ home_en.htm). The protocol was approved by the Institut Pasteur animal care and use committee. All experiments were conducted under enhanced biosafety level 3 conditions.

### Infection of mice and sample collection

Females C57BL/6J mice were purchased from the Centre d'Elevage R. Janvier (Le Genest Saint-Isle, France) and were used at about 8 weeks of age. Mice were lightly anesthetized with a mixture of ketamine and xylazine (60 mg/kg and 12 mg/kg respectively) and, unless otherwise stated, were inoculated intranasally with 200 TCID50 of virus in 30 µl PBS. Mice were observed daily for signs of morbidity and euthanized at different time points by cervical dislocation. To determine the lethal dose 50% in mice (MLD50), groups of 5 animals were infected with different dilutions of virus, weighed every other day and observed for signs of morbidity and death over 14 days. After euthanasia, lungs were aseptically removed and rinsed twice with PBS. One third of each lobe was taken and frozen in dry ice for further RNA extraction. The remaining two thirds were immediately fixed by immersion for 7 days in 10% neutral buffered formalin.

### Viruses and cells

The A/duck/Niger/2090/2006 virus was isolated by the National Influenza Center (Northern-France) at the Institut Pasteur in Paris (France) from duck stool collected in Niger during the 2006 African outbreak of H5N1 HPAIV, and passaged twice in MDCK cells. The seed was kindly provided by Prof. Sylvie Van Der Werf and further amplified in MDCK cells to constitute working stocks. MDCK (Madin-Darby Canine Kidney) cells were grown at 37°C under 5% CO2 in complete MEM [modified Eagle medium with 4.5 mg/mL l-glucose, 100 U/mL penicillin and 100 μg/mL streptomycin], supplemented with 5% heat-inactivated foetal calf serum (FCS). 293T (human kidney) cells were grown in complete DMEM [Dulbecco's modified Eagle medium with 4.5 mg/mL l-glucose, 100 U/mL penicillin and 100 μg/mL streptomycin], supplemented with 10% FCS.

### Generation of infectious recombinant viruses

The eight genomic segments of Influenza A/duck/Niger/2090/2006 (H5N1) were cloned into a bidirectional transcription plasmid derived from pHW2000 [51] to generate recombinant viruses. Sequences of the eight segments are available in supplementary information (Text S1). Briefly, viral RNA was isolated by using the QIAamp Viral RNA mini kit (Qiagen). The genomic RNA segments were transcribed into cDNA by using the Uni12 universal primer and further amplified by PCR using segment-specific primers containing BsmB1 (PB1, PB2, HA, NA, M, NS) or BfuA1 (PA, NP) restriction sites, as described by Hoffmann et al. [52]. After digestion with BsmB1 or BfuA1, the resulting DNA fragments were cloned at the BsmB1 site of the pRF483 plasmid. All plasmids' inserts were verified by the sequencing of positive clones using a Big Dye terminator sequencing kit and an automated sequencer (Applied Biosystems). A PB1-F2 knocked out mutant of the PB1 segment was generated by mutation of the initiation and the three in-frame ATG codons of the ORF into ACG, and of the TCA Ser12 codon into a TAA stop codon using the Quickchange site-directed mutagenesis kit (Stratagene, La Jolla, CA). The resulting mutations are silent in the PB1 ORF.

The method used for production of the recombinant A/duck/Niger/2090/2006 virus and its PB1-F2 knock-out mutant was adapted from previously described reverse genetics procedures [53]. Briefly, a subconfluent coculture of 293T and MDCK cells in a 35-mm dish was transfected with the eight pRF483 plasmids (0.5 μg of each), using 10 μl of Fugene HD transfection reagent (Roche). After 24 h of incubation at 35°C, the culture medium was removed, and the cells were further incubated for a 48 h-period at 35°C in DMEM supplemented with a reduced 2% concentration of FCS. The efficiency of reverse genetics was evidenced by a virus-induced cytopathic effect on MDCK cells and further confirmed by titration on MDCK cells. The working stocks of the recombinant viruses were prepared by two successive amplifications in MDCK cells at a multiplicity of infection (M.O.I.) of 10^−3^ for 3 days at 35°C in MEM. The mutations introduced were confirmed by reverse transcription, PCR amplification and sequencing of the PB1 segment of the wild-type and mutated generated viruses.

Virus titers were determined on MDCK cells by limiting dilution assays and determined according to the Reed and Muench method [54] as the reciprocal of the highest dilution of virus which induces cytopathic effect (CPE) in at least 5 out of 10 wells. They are expressed as 50 % of the tissue culture infectious dose per ml (TCID50/ml).

### Minigenome assay

To compare the activity of viral RNP complex in presence or absence of PB1-F2, we used a reporter plasmid pPolI-LUC which contains a firefly luciferase ORF flanked by the noncoding regions of the NA segment under the control of human polymerase I promoter. The minigenome studies were performed in 24-well plates. Briefly, 293T cells were transfected with 150 ng pPolI-LUC together with 50 ng of pRF483-PA-RT, 50 ng of pRF483-PB2-RT, 100 ng of pRF483-NP-RT, 50 ng of pRF483-PB1-RT or pRF483-PB1ΔF2-RT and 50 ng of the pRSV-βGal plasmid to control DNA uptake. The procedure used the Fugene HD transfection reagent (Promega) according to the manufacturer's instructions. Cells were washed twice with phosphate-buffered saline (PBS) and lysed in 100 μl of lysis buffer provided with the Firefly Luciferase Assay System (Promega). Firefly luciferase and ß-galactosidade activities were measured on 20 μl of cell extracts, using the Firefly luciferase substrate provided with the above-mentioned kit and a Centro luminometer (Berthold), and the β-Galactosidase Enzyme Assay System (Promega), respectively.

### Western blot

Cells were lysed in a buffer containing 500 mM NaCl, 50 mM Tris-HCl pH 8.0, 1% SDS and protease inhibitors (Roche). Cell lysates were separated by SDS-polyacrylamide gel electrophoresis (SDS-PAGE) and electrotransferred onto Immobilon-P membranes (Millipore). Membranes were blocked with phosphate-buffered saline containing 5% nonfat dry milk and 0.3% Tween 20, incubated with rabbit polyclonal anti-PB1-F2 (dilution 1∶5000) or anti-PB1 antibody (dilution 1∶200) and washed with phosphate-buffered saline containing 0.3% Tween 20. The PB1-F2 polyclonal antibody was produced in rabbit against the recombinant PB1-F2 protein from A/duck/Niger/2090/2006 (H5N1). The antibody (vC-19; Santa Cruz Biotechnology) is directed against the C-terminal part of PB1 and is able to recognize PB1 and N40. Finally, horseradish peroxidase-labeled anti-secondary antibody was used at a dilution of 1∶5000 for detection of bound primary antibody by enhanced chemiluminescence (Amersham).

### qRT-PCR

Total RNA was extracted from lung samples after homogenization in RLT buffer (RNeasy kit, Qiagen) using ceramic beads (lysing matrix D, MP biochemicals) and a FastPrep-24 instrument. Samples were clarified by low-speed centrifugation (3500g, 10 min.) and further processed according to manufacturer's instructions, including on-column DNase I digestion step to remove residual genomic DNA. RNA yield was determined by measuring the absorbance at 260 nm, and RNA integrity was checked using RNA 6000 serie II nano chips and the Agilent 2100 Bioanalyzer (Agilent Technologies). RNA quality was assessed using RNA integrity number. All samples had a 260/280 absorbance ratio around 2.1 and a RNA integrity number ≥6.5.

Reverse transcription, real-time PCR and relative quantitative evaluation of IFN-β trancripts were performed as described previously [17]. To assess vRNA levels, total RNA was reverse-transcribed with superscript II reverse transcriptase (Invitrogen) using the specific sense IAV M1 primer (5′-AGC AAA AGC AGG TAG ATA TTG-3′) and real-time PCR was performed in 20-μl reactions with Platinum Quantitative PCR SuperMix-UDG kit (Invitrogen) using the specific detection primer pairs (sense: 5′- CTT CTA ACC GAG GTC GAA ACG TA-3′; antisense: 5′-GGT GAC AGG ATC GGT CTT GTC TTT A–3′) and fluorogenic probe (5′ HEX-TCA GGC CCC CTC AAA GCC GAG-3′ BHQ-1). Absolute quantification of influenza M vRNA was done by using in vitro transcribed RNA standards prepared from the cloned M gene of A/Paris/650/04 and quantified spectrophotometrically.

### Histological analysis

After a one-week fixation in 10% neutral buffered formalin, lung tissues were transferred to 70% ethanol and stored at 4°C. Samples from each lung were embedded in paraffin; 5-μm sections were then cut and stained with hematoxylin and eosin (HE), and evaluated microscopically. Qualitative histological changes were described and when applicable were scored semi-quantitatively using a five grade scale from 0 to 4 (0: none, 1: minimal, 2: mild, 3: moderate, 4: marked), focusing on necrosis and inflammation (infiltration of neutrophils, lymphocytes and macrophages). The histological characterization of lesions was completed by an immunohistochemical detection of the viral antigens using polyclonal antibodies generated in rabbits against the A/Hong Kong/156/97 virus and the colorimetric immunohistochemistry LSAB kit procedure (DAKO, Glostrup, Denmark).

### Microarray experiments

Transcriptional profiling was performed using Agilent's Whole Mouse Genome Microarray Kit, 4×44 K (G4122F). Separate microarrays were run for each experimental sample (one sample per mouse and three or four mice per time point (Days post-infection (pi) 2 and 4: n = 3; mock and day pi 8: n = 4). Arrays were hybridized according to the manufacturer's instructions and as previously described [17]. A single color design was used to provide two comparisons: [uninfected/infected by WT Nig06 virus] and [uninfected/infected by ΔF2 Nig06 virus]. Identification of differentially expressed genes and functional investigation were done using GeneSpring GX 11 software (Agilent Technologies). For further analysis, data files were uploaded into the Ingenuity Pathways Analysis (IPA) software (Ingenuity Systems). Right-tailed Fisher's exact test was used to calculate a p-value determining the probability that each biological function and disease assigned to that data set is due to chance alone.

### Statistical analysis

Mice survival was compared using Kaplan-Meier analysis and log-rank test. Luciferase assays, histological scores and RT-PCR quantification are expressed as the mean ± standard error of the mean (SEM) of at least three separate replicates, and statistical analyzes were performed using the paired Student T test.

## Supporting Information

Text S1 **Sequences of Influenza A/duck/Niger/2090/2006 (H5N1) virus in Fasta format.**
(TXT)

Figure S1 **Alignment of PB1-F2 sequences from several H1N1 and H5N1 viral strains.** Location of key sequences and polymorphisms are indicated. CLUSTALW (http://www.ebi.ac.uk/Tools/msa/clustalw2/) was used to perform the alignment. The virus sequences used to make the alignment are: A/Puerto Rico/8/1934(H1N1) [PR8]; A/WSN/1933(H1N1) [WSN]; A/Brevig Mission/1/1918(H1N1) [1918]; A/duck/Niger/2090/2006(H5N1) [Nig06]; A/Viet Nam/1203/2004(H5N1) [VN04] and A/Hong Kong/156/97(H5N1) [HK97].
(PDF)
