# Supplementary material for: Kinetic Characterization of PB1-F2-Mediated Immunopathology during Highly Pathogenic Avian H5N1 Influenza Virus Infection
Source: PLoS One. 2013 Mar 1;8(3):e57894. doi: 10.1371/journal.pone.0057894 (PMC3585811; doi:10.1371/journal.pone.0057894)
Supplement: Figure S1 — Alignment of PB1-F2 sequences from several H1N1 and H5N1 viral strains. Location of key sequences and polymorphisms are indicated. CLUSTALW (http://www.ebi.ac.uk/Tools/msa/clustalw2/) was used to perform the alignment. The virus sequences used to make the alignment are: A/Puerto Rico/8/1934(H1N1) [PR8]; A/WSN/1933(H1N1) [WSN]; A/Brevig Mission/1/1918(H1N1) [1918]; A/duck/Niger/2090/2006(H5N1) [Nig06]; A/Viet Nam/1203/2004(H5N1) [VN04] and A/Hong Kong/156/97(H5N1) [HK97]. (PDF) [file pone.0057894.s002.pdf]

Figure S1. Leymarie *et al.*

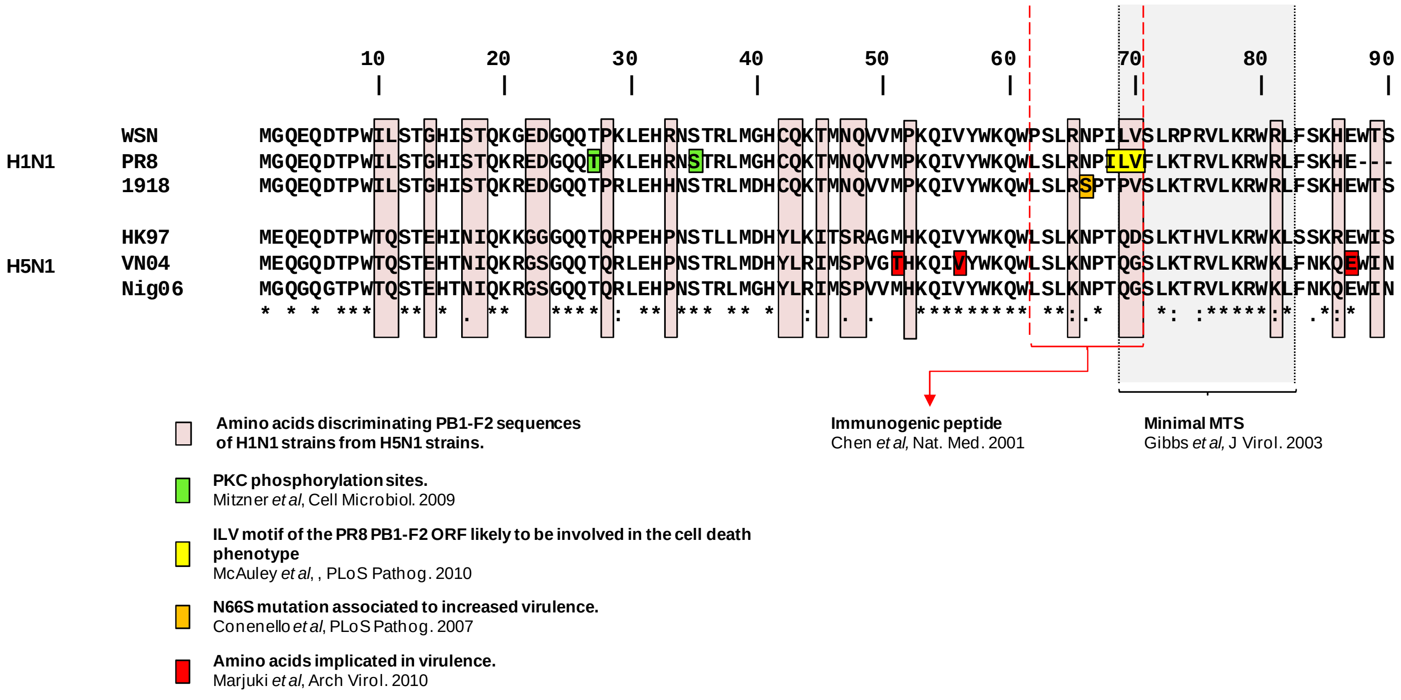

Supplemental Figure S1:

Alignment of PB1-F2 sequences from H1N1 and H5N1 viral strains. Location of key sequences and polymorphisms are indicated. CLUSTALW (http://www.ebi.ac.uk/Tools/msa/clustalw2/) was used to perform the alignment. The virus sequences used to make the alignment are : A/Puerto Rico/8/1934(H1N1) [PR8]; A/WSN/1933(H1N1) [WSN]; A/Brevig Mission/1/1918(H1N1) [1918]; A/duck/Niger/2090/2006(H5N1) [Nig06]; A/Viet Nam/1203/2004(H5N1) [VN04] and A/Hong Kong/156/97(H5N1) [HK97].
